
JOURNAL INFORMATION
==============================
NLM Title Abbreviation: BMC Proc
Journal ID: BMC Proc
NLM Journal ID: 101316936

BMC Proceedings

EISSN: 1753-6561
Publisher: BMC

ARTICLE INFORMATION
==============================
PMCID: PMC5615241
DOI: 10.1186/s12919-017-0074-9
Article ID: 74
Article version: 1
Subjects: Meeting Abstracts

Abstracts from the 1st International Symposium on Community Health Workers

Kampala, Uganda. 21–23 February 2017

Electronic publication date: 2017 Sep 19
Publication date: 2017
Volume: 11
Issue: Suppl 6
Issue sponsor: Publication charges for this supplement were funded by the Symposium. The Supplement Editors declare that they have no competing interests.
Electronic Location ID: 10
Copyright: © The Author(s). 2017
License: Open AccessThis article is distributed under the terms of the Creative Commons Attribution 4.0 International License (http://creativecommons.org/licenses/by/4.0/), which permits unrestricted use, distribution, and reproduction in any medium, provided you give appropriate credit to the original author(s) and the source, provide a link to the Creative Commons license, and indicate if changes were made. The Creative Commons Public Domain Dedication waiver (http://creativecommons.org/publicdomain/zero/1.0/) applies to the data made available in this article, unless otherwise stated.
License URL: https://creativecommons.org/licenses/by/4.0/

Conference name: 1st International Symposium on Community Health Workers
Conference Acronym: n.a.
Conference location: Kampala, Uganda
Conference date: 21-23 February 2017
issue-copyright-statement: © The Author(s) 2017

==============================
Download to read this full article text

Publisher’s Note

Springer Nature remains neutral with regard to jurisdictional claims in published maps and institutional affiliations.

